# Machine learning and nomogram for predicting severity of acute cholangitis: development and internal validation of a dual-framework model in a retrospective cohort study

**DOI:** 10.3389/fmed.2026.1936260

**Published:** 2026-10-06

**Authors:** Yun-Xiao Lyu, Bin Wang, Zhong Lyu

**Affiliations:** Department of Hepatobiliary Surgery, Affiliated Dongyang Hospital of Wenzhou Medical University, Dongyang, Zhejiang, China

**Keywords:** acute cholangitis, decision curve analysis, machine learning, nomogram, random forest, severity grading, Tokyo guidelines 2018

## Abstract

**Background:**

Accurate severity grading of acute cholangitis (AC) according to the Tokyo Guidelines 2018 (TG18) is essential for guiding biliary drainage decisions and intensive care allocation, yet bedside application of the multi-parameter grading system remains challenging. Existing prediction models are predominantly binary and lack interpretable clinical tools.

**Methods:**

This retrospective cohort study enrolled 1,918 consecutive patients with acute cholangitis graded by TG18 at a single tertiary hepatobiliary centre (2021–2025). Random forest (RF), XGBoost, and support vector machine (SVM) were trained on seven LASSO-selected variables for three-class Grade I/II/III prediction. A separate eight-variable logistic regression nomogram distinguished Grade I from Grade II/III. Models were developed on a 70% training set (*n* = 1,342) and evaluated on a 30% held-out internal test set (*n* = 576) using bootstrap confidence intervals.

**Results:**

RF achieved macro-AUC 0.84 (95% CI: 0.81–0.87), overall accuracy 72.2% and balanced accuracy 64.9%; XGBoost performed comparably (macro-AUC 0.84, accuracy 71.9%). Per-class recall for RF was 0.83 (Grade I), 0.56 (Grade II) and 0.56 (Grade III), indicating that almost half of Grade II cases were not correctly identified. The nomogram yielded AUC 0.85 (0.82–0.88), sensitivity 0.72, specificity 0.82, and negative predictive value 0.82 at the Youden reference threshold of 0.47; operating characteristics at other thresholds, including a sensitivity-weighted rule-out threshold, are reported in the Supplementary Material. Decision curve analysis suggested potential net benefit across the evaluated range of threshold probabilities. SHAP analysis identified platelet count, age, and serum albumin as the top predictors.

**Conclusions:**

A dual-framework combining ML-based three-class severity grading and a nomogram for binary triage may provide complementary support for standardising TG18 severity assessment. Both frameworks predict the TG18 grade itself rather than downstream clinical outcomes, and Grade II recognition remains limited; they should therefore be regarded as adjuncts to—not replacements for—TG18 assessment. Prospective multicentre validation against clinical outcomes is required before any clinical use.

## Introduction

Acute cholangitis (AC) is a potentially life-threatening biliary tract infection characterised by bacterial growth in bile under conditions of biliary obstruction. The Tokyo Guidelines 2018 (TG18) provide the current standard for severity classification, stratifying patients into three grades: mild (Grade I), moderate (Grade II), and severe (Grade III), with Grade III associated with organ dysfunction and a reported 30-day mortality of 5.1%, against 2.6% for Grade II and 1.2% for Grade I (1, 2). Prompt and accurate severity assessment is essential for selecting antimicrobial therapy (3) and for deciding the timing of biliary drainage and escalation of care (4).

Despite widespread adoption of TG18, bedside application remains challenging. Severity grading requires integration of multiple clinical and laboratory parameters across organ systems, and a prospective multinational audit has documented that adherence to the Tokyo Guidelines in routine practice is variable (5). The boundary between Grade I and Grade II is particularly demanding, because it depends on parameters that may not all be documented at presentation (1, 6). Misclassification at this boundary is clinically consequential, since it determines whether biliary drainage is planned early.

Machine learning (ML) algorithms offer a promising approach to multi-variable clinical prediction, with demonstrated performance across a range of clinical tasks (7) and, in a related biliary-pancreatic emergency, in predicting severe acute pancreatitis on admission (8). However, most existing AC prediction models dichotomise severity (severe vs. non-severe), collapsing Grade I and Grade II into a single non-severe category, and therefore do not address the clinically important distinction between them (9, 10). Furthermore, ML-derived models are rarely accompanied by interpretable bedside tools suitable for non-specialist settings.

The objective of this study was to develop and internally validate a dual-framework system: (i) an ML three-class severity prediction model and (ii) a logistic regression nomogram for binary triage (Grade I vs. Grade II/III), with the aim of providing complementary decision-support tools across different clinical settings. Both frameworks were conceived as tools to operationalise and standardise TG18 severity assessment from a parsimonious set of routinely available admission variables, rather than as predictors of an outcome that is clinically or informationally independent of TG18. The binary cut-point (Grade I versus Grade II/III) was prespecified because TG18 recommends early biliary drainage for Grade II and urgent drainage with organ support for Grade III, whereas Grade I is initially managed with antibiotics and supportive care (1, 6); this boundary therefore represents the principal point at which management escalates.

## Materials and methods

### Study design and patients

This retrospective cohort study was conducted at the Affiliated Dongyang Hospital of Wenzhou Medical University, a tertiary hepatobiliary referral centre in Dongyang, Zhejiang, China. Electronic medical records were reviewed for consecutive patients admitted with a diagnosis of AC between January 2021 and December 2025. All patients were graded by TG18 criteria within 24 h of admission by the attending hepatobiliary surgery team.

Inclusion criteria: (i) a definite diagnosis of AC according to the TG18 diagnostic criteria, requiring all three domains to be satisfied—A, systemic inflammation (fever and/or shaking chills, or abnormal white blood cell count or C-reactive protein); B, cholestasis (jaundice or abnormal liver function tests); and C, imaging evidence of biliary dilatation or of an aetiology such as stricture, stone or stent, on ultrasonography, computed tomography or magnetic resonance cholangiopancreatography (1); (ii) TG18 severity grading recorded within 24 h of admission; (iii) first-ever AC episode. Patients meeting only the TG18 criteria for a suspected diagnosis (domain A plus either B or C) were not included. Classical presentations (Charcot's triad, Reynolds’ pentad) were recorded but were not required for inclusion. Exclusion criteria: (i) recurrent AC; (ii) concurrent infectious or inflammatory conditions that could confound laboratory values; (iii) unavailability of the admission data required to assign the TG18 severity grade or of the core admission laboratory panels (see Missing Data and Statistical Software). A total of 1,918 patients met the eligibility criteria. Data were randomly split into a training set (70%, *n* = 1,342) and a held-out internal test set (30%, *n* = 576), stratified by TG18 grade (random seed = 42). The split was performed before any preprocessing, and the test set was used only for final evaluation.

This study was reviewed by a statistician, and statistical methods were considered appropriate for the study design. This manuscript was prepared and reported in accordance with the TRIPOD + AI statement for clinical prediction models developed using regression or machine learning methods (11), which updates the original TRIPOD statement (12). A completed TRIPOD + AI checklist is provided as Supplementary Material, together with a STROBE checklist for the observational cohort component (13).

### Variables and outcome

The primary outcome was TG18 severity grade (Grade I, II, or III). Severity was assigned by the attending hepatobiliary surgery team within 24 h of admission, using the same admission laboratory values that were subsequently offered to the models as predictors. The models were therefore designed to reproduce a formal TG18 assessment from admission data, not to predict an outcome that is temporally or informationally separable from the predictors; the implications of this shared information are considered explicitly in the Discussion. Eighteen continuous candidate predictors were prespecified across six clinical domains reflecting the physiology captured by TG18: demographic—age; hepatobiliary and cholestatic—total bilirubin (TB), direct bilirubin (DB), albumin (ALB), alanine aminotransferase (ALT), aspartate aminotransferase (AST), alkaline phosphatase (ALP), gamma-glutamyl transferase (GGT); systemic inflammation—white blood cell count (WBC), neutrophil count (NEU), C-reactive protein (CRP); coagulation—prothrombin time (PT), international normalised ratio (INR), platelet count (PLT); renal function—serum creatinine (SCr), blood urea nitrogen (BUN); and metabolic/nutritional—total cholesterol (TC), low-density lipoprotein cholesterol (LDL). All predictors were the first values recorded after presentation, obtained within 24 h of admission and before antibiotic administration. Procalcitonin was excluded *a priori* because 36.6% of values were missing, exceeding the prespecified 20% threshold for reliable imputation. Sex, comorbidities, aetiology and hospital length of stay were tabulated for baseline description only (Table 1); none was a candidate predictor and none entered any model.

**Table 1:** Baseline characteristics of the study cohort stratified by TG18 severity grade.

Variable	Overall (*n* = 1,918)	Grade I (*n* = 1,163)	Grade II (*n* = 391)	Grade III (*n* = 364)	*P* value
Demographics					
Age (years), mean ± SD	67.7 ± 15.7	63.0 ± 15.8	75.2 ± 12.9	74.4 ± 12.4	<0.001
Male sex, *n* (%)	1,063 (55.4)	601 (51.7)	226 (57.8)	236 (64.8)	<0.001
Hospital stay (days), median (IQR)	9.0 (6.0–14.0)	8.0 (6.0–12.0)	10.0 (7.0–16.0)	11.0 (8.0–17.0)	<0.001
Comorbidities, *n* (%)					<0.001
Hypertension	766 (39.9)	408 (35.1)	187 (47.8)	171 (47.0)	
Diabetes mellitus	263 (13.7)	133 (11.4)	57 (14.6)	73 (20.1)	
Coronary artery disease	199 (10.4)	101 (8.7)	42 (10.7)	56 (15.4)	
COPD	56 (2.9)	21 (1.8)	17 (4.3)	18 (4.9)	
Malignancy	400 (20.9)	204 (17.5)	109 (27.9)	87 (23.9)	
Renal insufficiency	69 (3.6)	10 (0.9)	14 (3.6)	45 (12.4)	
Liver cirrhosis	90 (4.7)	35 (3.0)	35 (9.0)	20 (5.5)	
Laboratory values, median (IQR)					
Total bilirubin (*μ*mol/L)	34.3 (16.0–67.5)	25.8 (14.6–55.7)	54.1 (22.6–95.3)	46.2 (22.8–79.2)	<0.001
Direct bilirubin (μmol/L)	20.6 (6.8–50.6)	13.5 (5.5–38.6)	40.6 (12.8–70.3)	33.9 (10.9–62.6)	<0.001
ALT (U/L)	92.5 (30.0–223.0)	98.0 (26.0–250.0)	94.0 (38.0–195.5)	74.0 (31.8–189.8)	0.326
AST (U/L)	74.5 (30.0–183.0)	68.0 (26.0–187.5)	88.0 (43.5–172.0)	77.0 (35.0–192.5)	0.013
ALP (U/L)	156.0 (102.0–247.0)	150.0 (98.0–235.5)	189.0 (120.5–313.5)	142.5 (99.8–234.2)	<0.001
GGT (U/L)	239.0 (77.2–453.0)	237.0 (60.0–470.0)	270.0 (125.5–493.5)	207.5 (86.8–370.0)	0.004
Albumin (g/L)	36.9 (32.7–41.1)	38.7 (35.3–42.5)	34.1 (30.1–38.0)	32.2 (28.4–36.8)	<0.001
Hemoglobin (g/L)	130.0 (116.5–142.0)	132.0 (121.0–144.0)	125.0 (111.2–139.0)	126.0 (112.0–140.0)	<0.001
WBC (×10⁹/L)	8.2 (5.8–11.4)	7.3 (5.5–9.9)	11.3 (7.3–14.1)	9.2 (6.2–12.8)	<0.001
Neutrophil count (×10⁹/L)	6.5 (4.0–9.7)	5.4 (3.5–8.2)	9.6 (5.7–12.5)	8.0 (4.9–11.1)	<0.001
Lymphocyte count (×10⁹/L)	0.9 (0.6–1.4)	1.1 (0.8–1.6)	0.7 (0.4–1.1)	0.7 (0.4–1.1)	<0.001
Platelet count (×10⁹/L)	185.0 (137.0–235.0)	203.0 (158.0–249.0)	178.5 (140.0–230.8)	120.0 (85.0–180.2)	<0.001
PT (s)	13.5 (12.8–14.5)	13.2 (12.6–13.9)	14.1 (13.3–15.1)	14.4 (13.3–16.0)	<0.001
INR	1.1 (1.0–1.1)	1.0 (1.0–1.1)	1.1 (1.0–1.2)	1.1 (1.0–1.3)	<0.001
Serum creatinine (μmol/L)	67.0 (54.0–84.0)	63.0 (52.5–78.0)	70.0 (57.0–88.0)	78.5 (61.0–114.0)	<0.001
BUN (mmol/L)	5.3 (4.0–6.8)	4.8 (3.8–6.1)	5.8 (4.5–7.4)	6.6 (4.8–10.0)	<0.001
CRP (mg/L)	13.6 (2.2–59.0)	6.2 (1.3–28.9)	44.9 (11.1–93.8)	43.5 (6.7–105.2)	<0.001
Triglycerides (mmol/L)	1.0 (0.7–1.5)	1.1 (0.8–1.5)	1.0 (0.6–1.4)	0.9 (0.6–1.5)	0.003
Total cholesterol (mmol/L)	3.9 (3.1–4.6)	4.2 (3.5–4.9)	3.5 (3.0–4.4)	3.1 (2.4–3.8)	<0.001
Amylase (U/L)	63.0 (44.0–93.0)	64.2 (48.0–90.0)	60.0 (39.0–97.0)	61.0 (38.0–92.6)	0.005
Calcium (mmol/L)	2.2 (2.1–2.3)	2.2 (2.1–2.3)	2.2 (2.1–2.3)	2.2 (2.1–2.3)	<0.001

Data are presented as mean ± standard deviation for normally distributed continuous variables, median (interquartile range) for non-normally distributed continuous variables, and *n* (%) for categorical variables.

*P* values were derived from the Kruskal–Wallis test for continuous variables and the Pearson *χ*² test or Fisher exact test for categorical variables, as appropriate. A single grouped *P* value is reported for comorbidities and reflects overall heterogeneity across severity grades.

ALP, alkaline phosphatase; ALT, alanine aminotransferase; AST, aspartate aminotransferase; BUN, blood urea nitrogen; COPD, chronic obstructive pulmonary disease; CRP, C-reactive protein; GGT, gamma-glutamyl transferase; INR, international normalized ratio; IQR, interquartile range; PT, prothrombin time; SD, standard deviation; TG18, Tokyo Guidelines 2018; WBC, white blood cell count.

### Variable selection

LASSO regularisation (glmnet implementation, penalty parameter *λ* selected by 10-fold cross-validation) was applied to the training set to identify the most informative features for each framework. Candidate variables first underwent univariate Kruskal–Wallis screening across the three grades, performed on the training set alone so that no information from the held-out test set entered variable selection, using a deliberately liberal threshold of *P* < 0.10 (Supplementary Table S1; Supplementary Figure S1). Two candidates failed screening and were excluded—ALT (H = 2.7, *P* = 0.256) and AST (H = 4.0, *P* = 0.138)—leaving 16 variables. These then underwent collinearity assessment on the training set (Spearman |r| ≥ 0.80; Supplementary Table S2; Supplementary Figure S2). Where a pair exceeded that threshold, the member in more routine clinical use was retained—total rather than direct bilirubin, INR rather than prothrombin time, and neutrophil rather than total white cell count—leaving 12 variables. LASSO regularisation with tenfold cross-validation was then applied (14) (Supplementary Figure S3). The final models retained the variables with the largest LASSO coefficients that are also available as routine admission tests, so that the bedside tool would remain short: seven variables for the three-class ML framework (ALB, INR, age, PLT, NEU, TB, SCr) and eight for the binary nomogram (the same seven plus CRP). Because this last restriction was made for parsimony rather than by a statistical rule, we report in the Supplementary Material the full set of variables that LASSO itself retains (10 under the one-standard-error rule and 12 at the cross-validated optimum) together with the performance of models built on them (Supplementary Table S11). The rationale for screening and collinearity filtering before penalised regression was to remove redundant assays measuring the same biological construct (for example TB and DB, WBC and NEU, PT and INR) so that the resulting bedside tool would remain parsimonious. Because univariate pre-screening can in principle discard predictors that are informative only in a multivariable context, a sensitivity analysis applying LASSO directly to all 18 prespecified candidates, without screening or collinearity filtering, is reported in Supplementary Table S7.

### Machine learning models

Three supervised classifiers were trained: random forest (15), XGBoost (16), and a support vector machine with a radial basis function kernel. Hyperparameters were selected by exhaustive grid search under five-fold stratified cross-validation of the training set, optimising macro one-vs.-rest AUC; the complete search space and the final selected values are reported in Supplementary Table S3. Class imbalance (Grade I: Grade II: Grade III ≈ 60:20:20) was addressed by the synthetic minority oversampling technique (SMOTE) (17). SMOTE was applied to the training set only; the held-out test set was never resampled, so the test-set estimates reported below cannot be inflated by synthetic observations. In the primary analysis, median imputation, standardisation, variable selection and SMOTE were each fitted on the whole training set before cross-validation, which was used only to select hyperparameters; this can in principle render hyperparameter selection mildly optimistic. To quantify that possibility, two confirmatory analyses were performed in which imputation, standardisation, SMOTE and the classifier were encapsulated in a single pipeline and refitted independently within every cross-validation training fold—first with the seven selected variables held fixed, and second with the LASSO selection step also refitted inside each fold, applied to the 16 variables retained after screening. The univariate screening and collinearity filtering that preceded LASSO were performed once on the whole training set and were not repeated within folds, so these analyses bound rather than eliminate the potential for optimism (Supplementary Table S8). Model performance was evaluated on the held-out internal test set using macro-AUC and per-class AUC (one-vs.-rest), accuracy, balanced accuracy, and per-class precision, recall and F1-score for the multiclass framework, and sensitivity, specificity and predictive values for the nomogram. Bootstrap 95% confidence intervals (1,000 iterations) were computed for the macro-AUC of each machine learning model and for the nomogram AUC; the remaining measures are reported as point estimates. Shapley Additive Explanations (SHAP) values were computed to quantify predictor importance (18).

### Nomogram development

A binary logistic regression model was developed on the training set to distinguish Grade I from Grade II/III (clinically actionable triage threshold for biliary drainage). Eight LASSO-selected variables were included. A reference probability threshold was derived from the Youden index on the training set. Because sensitivity and specificity carry unequal clinical weight when the purpose is to avoid missing moderate-to-severe disease, operating characteristics were additionally computed across a range of candidate thresholds (Supplementary Table S9). The nomogram was constructed with the rms R package (R version 4.3.2). Calibration was assessed by the Hosmer–Lemeshow goodness-of-fit test and calibration curves. Decision curve analysis (DCA) was used to estimate the theoretical net benefit of the nomogram relative to treat-all and treat-none strategies across threshold probabilities from 0 to 1 (19). DCA quantifies potential net benefit under assumptions about the relative cost of false-positive and false-negative classifications; it does not demonstrate realised clinical benefit.

### Missing data and statistical software

Among the 18 candidate predictors in the 1,918 included patients, 2.5% of all feature values were missing. Missingness was below 5% for every candidate except total cholesterol and low-density lipoprotein cholesterol (14.6% each), neither of which was retained in any final model; C-reactive protein was missing in 4.6% of patients, INR and PT in 2.5%, and all remaining candidates in less than 2.0% (Supplementary Table S10). Missing values were imputed by median imputation; imputation parameters were estimated from the training set only and applied unchanged to the test set to prevent leakage. No patient was excluded on the basis of missingness in these candidate variables; exclusion criterion (iii) applied only to patients in whom the TG18 severity grade could not be assigned or in whom the core admission laboratory panels had not been obtained. Analyses were performed in Python 3.10 (scikit-learn 1.2, shap 0.41, xgboost 1.7) and R 4.3.2. Statistical significance was set at *α* = 0.05.

## Results

### Patient characteristics

A total of 1,918 patients were included: 1,163 (60.6%) Grade I, 391 (20.4%) Grade II, and 364 (18.9%) Grade III. The training set (70%, *n* = 1,342) and held-out internal test set (30%, *n* = 576) were balanced in grade distribution. The analysis workflow is summarised in Figure 1. Baseline characteristics by TG18 grade are summarised in Table 1, and the distributions of key laboratory variables are shown in Figure 2. Grade III patients were significantly older, had higher WBC, neutrophil count, total bilirubin, C-reactive protein, INR and serum creatinine, and lower albumin and platelet count than Grade I patients (all *P* < 0.001).

**Figure 1:**
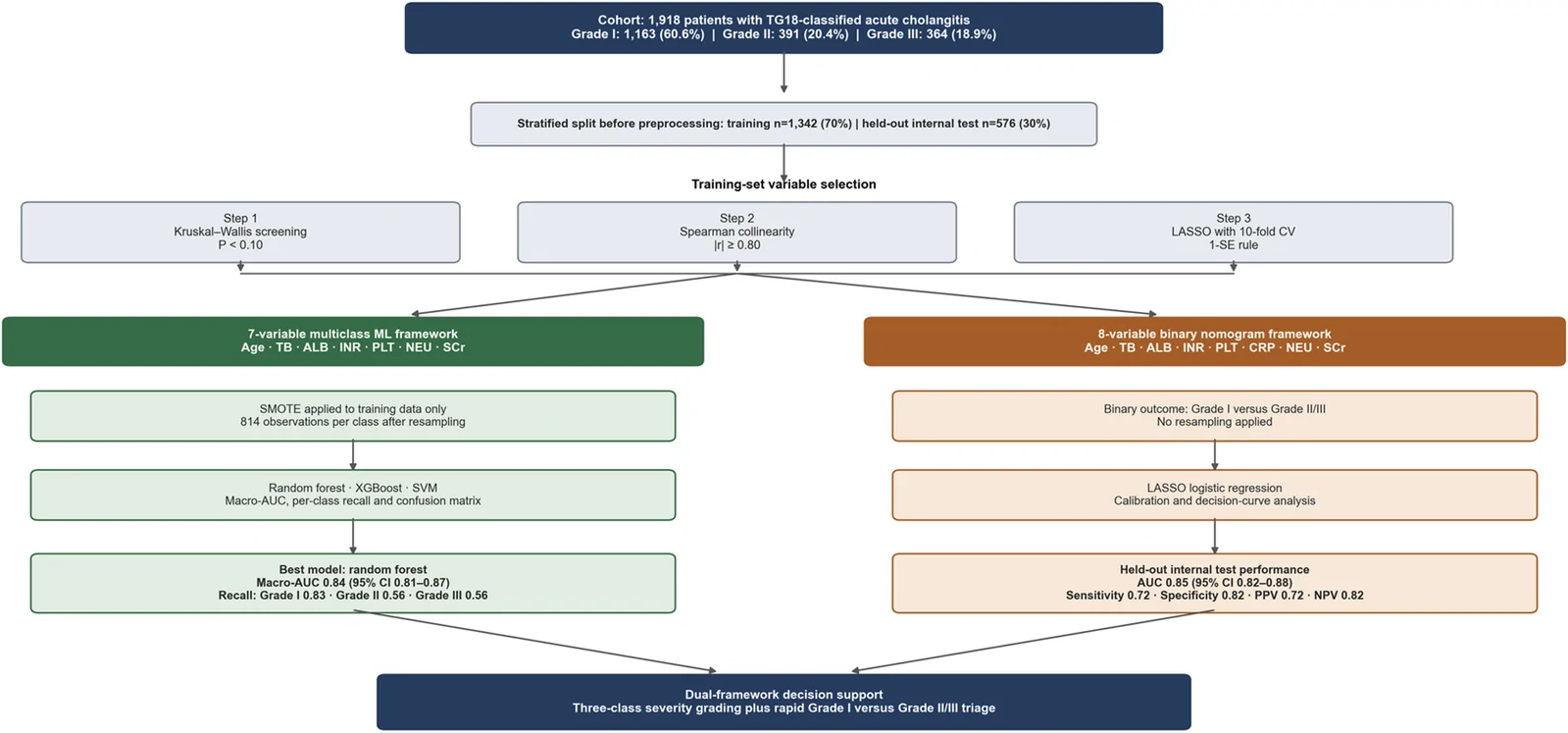
Analysis workflow. Composition of the analysed cohort (*n* = 1,918) by TG18 grade; the stratified 70/30 split into a training set (*n* = 1,342) and a held-out internal test set (*n* = 576), performed before any preprocessing; the three-step training-set variable selection procedure; and the two modelling frameworks with their principal performance results. Patient screening and exclusions are reported in the Results.

**Figure 2:**
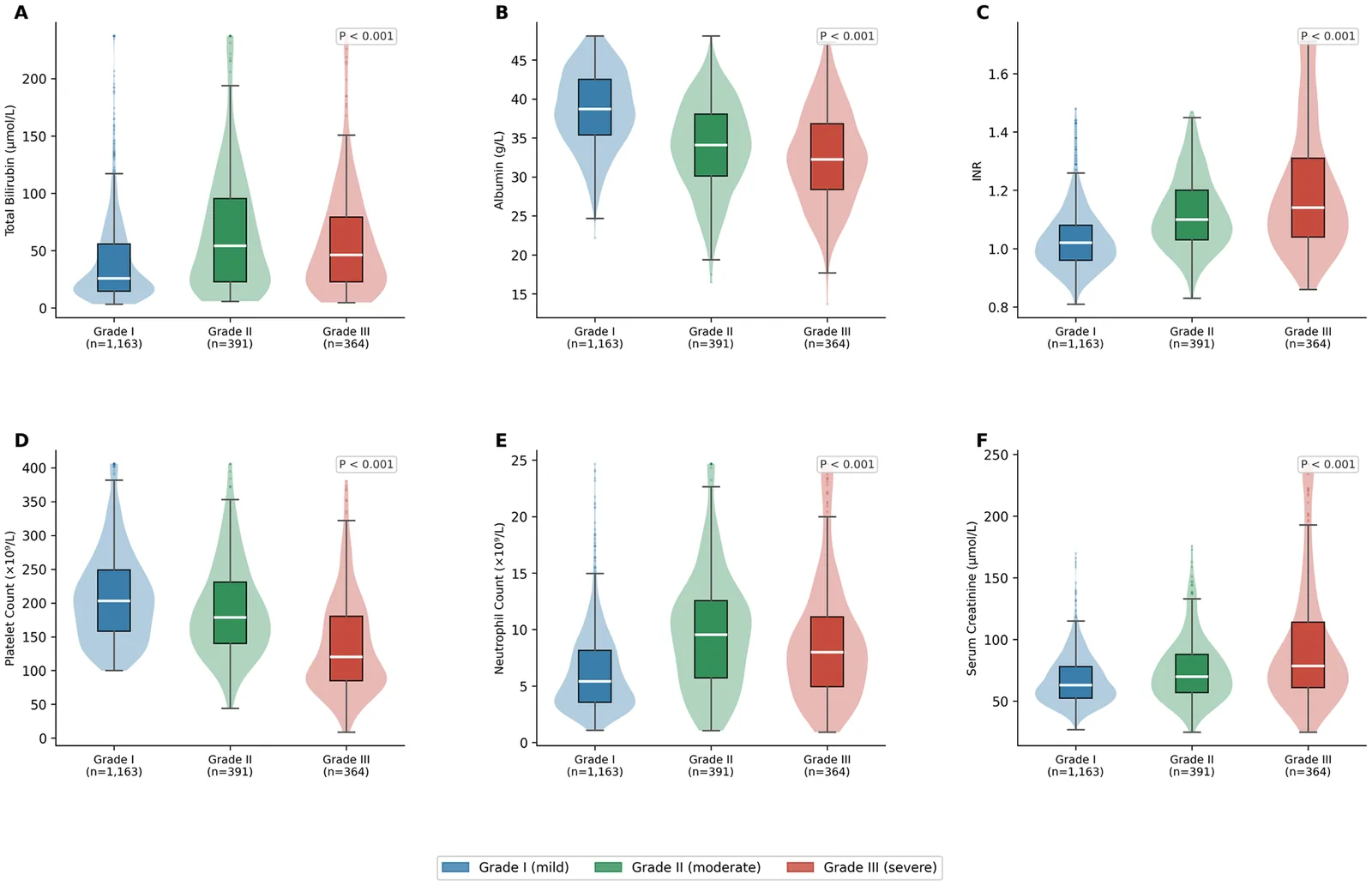
Distributions of key laboratory variables by TG18 severity grade. Violin plots with embedded box plots for total bilirubin **(A)**, albumin **(B)**, international normalised ratio **(C)**, platelet count **(D)**, neutrophil count **(E)**, and serum creatinine **(F)** in Grade I (*n* = 1,163), Grade II (*n* = 391), and Grade III (*n* = 364). *P* values are from Kruskal–Wallis tests across the three grades.

### Machine learning model performance

On the held-out test set, RF achieved the highest performance: macro-AUC 0.84 (95% CI: 0.81–0.87), accuracy 72.2%, per-class AUC for Grade III 0.89 (0.86–0.92). XGBoost performed comparably (macro-AUC 0.84 [0.81–0.87], accuracy 71.9%); SVM was slightly inferior (macro-AUC 0.80 [0.77–0.84], accuracy 65.5%). Balanced accuracy, which weights all three grades equally, was 0.649 for RF, 0.634 for XGBoost and 0.602 for SVM. Per-class discrimination, precision, recall, F1-score and support for all three models are presented in Table 2, with the corresponding one-vs.-rest AUC values also given in Supplementary Table S4 and the full metric set in Supplementary Table S5. Per-grade ROC curves and confusion matrices are shown in Figure 3. For RF, recall was 0.83 for Grade I, 0.56 for Grade II and 0.56 for Grade III. Of the 118 Grade II patients in the test set, 66 (56%) were correctly classified, 29 (25%) were assigned to Grade I and 23 (19%) to Grade III. SHAP analysis identified PLT, age, and ALB as the three most influential predictors across all three grades (Figure 4).

**Table 2:** Per-class and overall performance of the machine learning models for three-class TG18 severity prediction on the held-out internal test set (*n* = 576).

Model	Grade	AUC (OvR)	Precision	Recall	F1-score	Support (n)	Macro AUC	Accuracy	Balanced accuracy	Macro F1
Random forest	Grade I	0.891	0.86	0.83	0.84	349	0.843	72.2%	0.649	0.65
	Grade II	0.797	0.47	0.56	0.51	118				
	Grade III	0.839	0.61	0.56	0.58	109				
XGBoost	Grade I	0.892	0.84	0.84	0.84	349	0.838	71.9%	0.634	0.64
	Grade II	0.804	0.47	0.52	0.49	118				
	Grade III	0.817	0.60	0.54	0.57	109				
SVM (RBF)	Grade I	0.866	0.87	0.73	0.80	349	0.804	65.5%	0.602	0.59
	Grade II	0.747	0.38	0.52	0.44	118				
	Grade III	0.801	0.49	0.56	0.52	109				

Models were trained on the SMOTE-balanced training set (*n* = 1,342) using seven variables (albumin, INR, age, platelet count, neutrophil count, total bilirubin, serum creatinine) and evaluated on the held-out internal test set (*n* = 576), which was never resampled.

Macro AUC was computed by a one-vs.-rest strategy; bootstrap 95% CIs (1,000 iterations, seed = 42) were: random forest 0.812–0.870, XGBoost 0.807–0.866, SVM 0.772–0.836.

Balanced accuracy is the unweighted mean of per-class recall and is reported because overall accuracy is dominated by the majority Grade I class. Recall for Grade II was 0.56 for random forest, indicating that almost half of moderate cases were not correctly identified; the corresponding confusion matrices are shown in Figure 3.

Absolute confusion-matrix counts (rows = true grade, columns = predicted grade). Random forest—Grade I: 289/44/16; Grade II: 29/66/23; Grade III: 17/31/61. XGBoost—Grade I: 294/39/16; Grade II: 33/61/24; Grade III: 21/29/59. SVM—Grade I: 255/61/33; Grade II: 26/61/31; Grade III: 11/37/61.

AUC, area under the receiver operating characteristic curve; INR, international normalised ratio; OvR, one-vs.-rest; RBF, radial basis function; SMOTE, synthetic minority oversampling technique; SVM, support vector machine; TG18, Tokyo Guidelines 2018.

**Figure 3:**
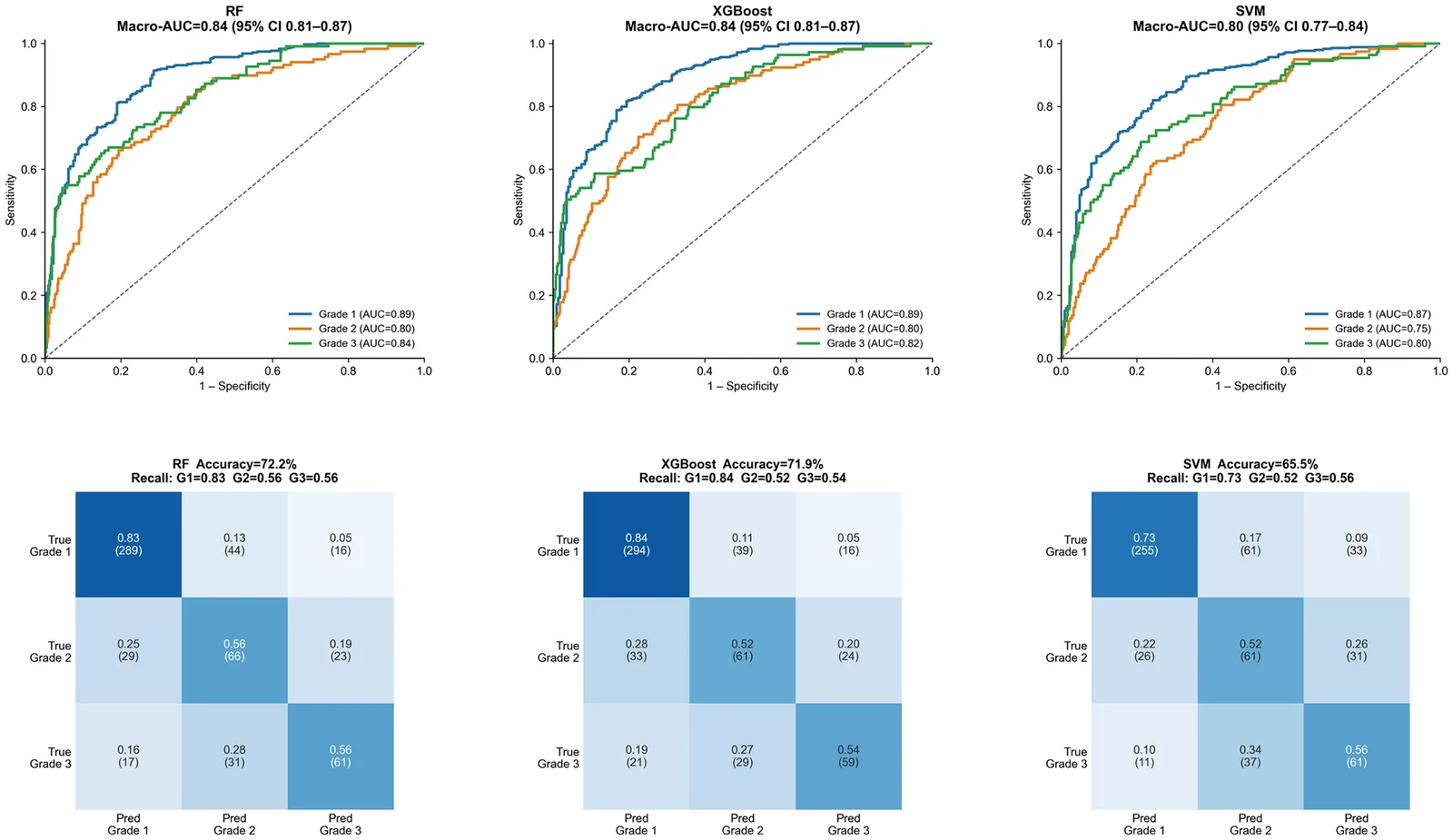
Machine learning model performance on the held-out internal test set (*n* = 576). Upper row: one-vs.-rest receiver operating characteristic (ROC) curves for Grade I, Grade II, and Grade III for random forest (RF), XGBoost, and support vector machine (SVM). Lower row: row-normalised confusion matrices; the diagonal gives per-class recall, and off-diagonal cells show the direction of misclassification.

**Figure 4:**
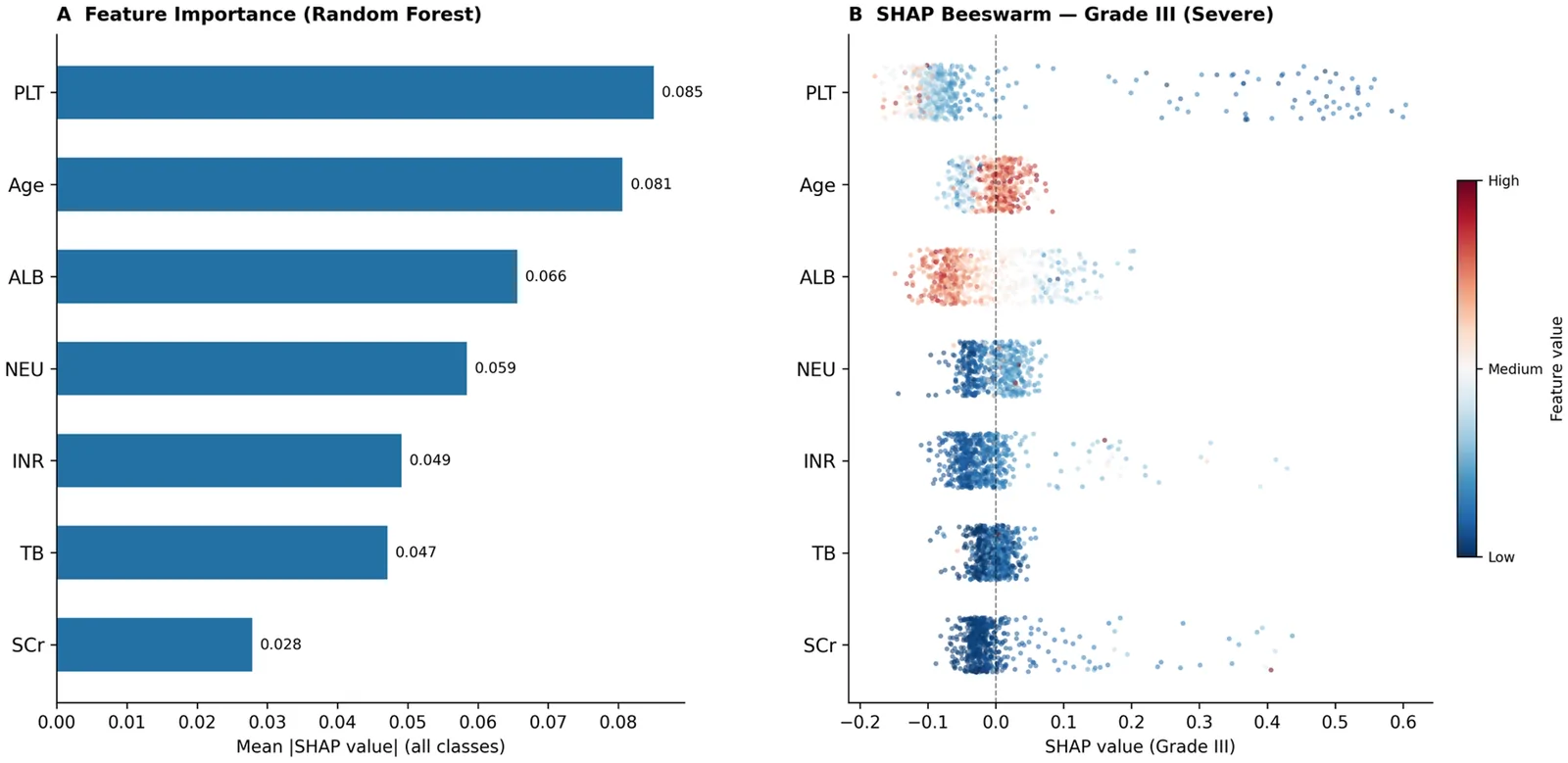
SHAP interpretability analysis for the random forest model. **(A)** Mean absolute SHAP value per predictor, averaged across the three grades. **(B)** SHAP beeswarm plot; each dot represents one patient, horizontal position reflects the SHAP value (impact on model output), and colour indicates the feature value (red = high, blue = low). The three most influential predictors were platelet count, age, and albumin.

Two prespecified sensitivity analyses supported the robustness of the development strategy. First, applying LASSO directly to all 18 prespecified candidates without univariate screening or collinearity filtering selected 15 variables for the binary framework, with a test-set AUC of 0.86 compared with 0.85 for the parsimonious eight-variable nomogram, and 13 variables for the multiclass framework, with a random forest test-set macro-AUC of 0.85 compared with 0.84; the additional variables carried only marginal coefficients (Supplementary Table S7). Second, encapsulating imputation, standardisation, SMOTE and the classifier in a single pipeline refitted within every cross-validation fold yielded cross-validated macro-AUCs of 0.873 (RF), 0.872 (XGBoost) and 0.841 (SVM), with held-out test macro-AUCs of 0.846, 0.847 and 0.821; additionally refitting the LASSO selection step within each fold gave test macro-AUCs of 0.851, 0.858 and 0.822. These values match or marginally exceed the primary analysis, indicating that the resampling, the hyperparameter search and the LASSO step contributed no material optimism. The univariate screening and collinearity filtering that precede them were performed once on the training set and were not repeated within folds, so these analyses bound rather than exclude optimism arising from the earliest selection steps (Supplementary Table S8).

### Nomogram performance

The binary logistic regression nomogram (Grade I vs. Grade II/III; Figure 5) achieved AUC 0.85 (95% CI: 0.82–0.88) on the held-out internal test set (Table 3; Figure 6), with sensitivity 0.72, specificity 0.82, positive predictive value 0.72, and negative predictive value 0.82 at the Youden reference threshold of 0.47. Operating characteristics at alternative thresholds are reported in Supplementary Table S9: at a sensitivity-weighted rule-out threshold of 0.20, sensitivity was 0.95 and negative predictive value 0.94, so that 5 of every 100 patients with Grade II/III disease would be missed rather than 29 at the Youden threshold, at the cost of specificity (0.48). Calibration was satisfactory (Hosmer–Lemeshow *P* = 0.34). Nomogram variable coefficients, odds ratios, and 95% CIs are presented in Supplementary Table S6. DCA indicated potential net benefit relative to treat-all and treat-none strategies across the evaluated range of threshold probabilities (0.15–0.80) (Figure 6).

**Figure 5:**
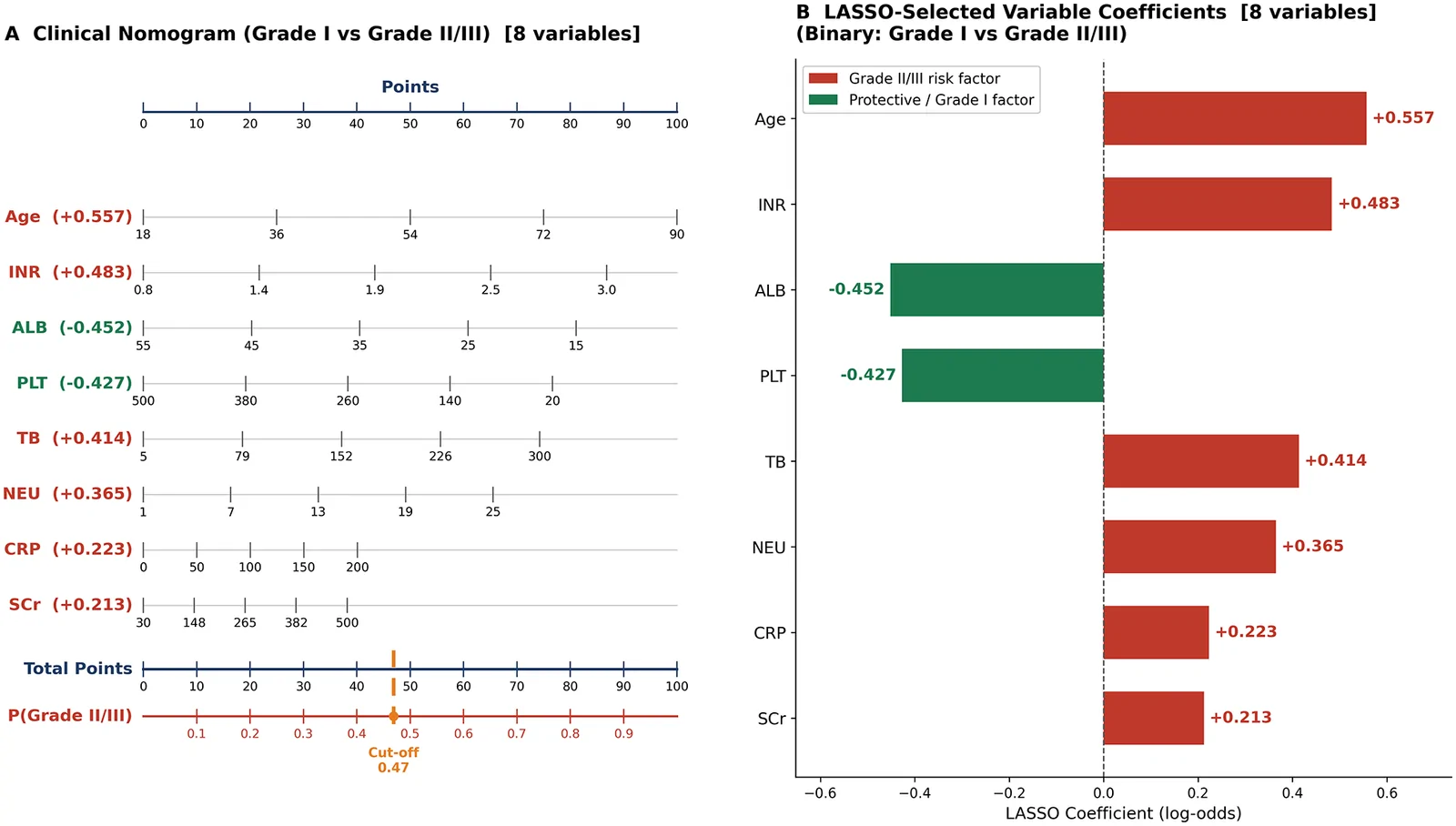
Nomogram for binary triage of acute cholangitis (grade I vs. Grade II/III). **(A)** Clinical nomogram: each variable is assigned a score on the Points axis, and the summed Total Points are converted to the predicted probability of Grade II/III on the lower axis. **(B)** LASSO-selected variable coefficients, expressed as log-odds per one standard deviation increase.

**Table 3:** Discrimination and operating characteristics of the LASSO-based clinical nomogram for binary triage (grade I versus grade II/III) on the held-out internal test set (*n* = 576).

Dataset	AUC	95% CI	Threshold	Sensitivity	Specificity	PPV	NPV
Training set (*n* = 1,342)	0.87	—	—	—	—	—	—
Held-out internal test set (*n* = 576)	0.85	0.82–0.88	0.47	0.72	0.82	0.72	0.82

Binary classification: Grade I (mild) versus Grade II/III (moderate–severe) according to TG18.

The reference threshold of 0.47 was derived from the Youden index on the training set and applied without modification to the held-out internal test set. Because sensitivity and specificity carry unequal clinical weight in triage, operating characteristics at alternative thresholds—including a sensitivity-weighted rule-out threshold of 0.20 (sensitivity 0.95, NPV 0.94)—are reported in Supplementary Table S9.

Training-set AUC is shown for reference only; the 95% CI and operating characteristics are reported for the test set alone, since apparent training-set performance is subject to optimism.

Bootstrap 95% CI for the test-set AUC was derived from 1,000 iterations (seed = 42).

AUC, area under the receiver operating characteristic curve; CI, confidence interval; LASSO, least absolute shrinkage and selection operator; NPV, negative predictive value; PPV, positive predictive value; TG18, Tokyo Guidelines 2018.

**Figure 6:**
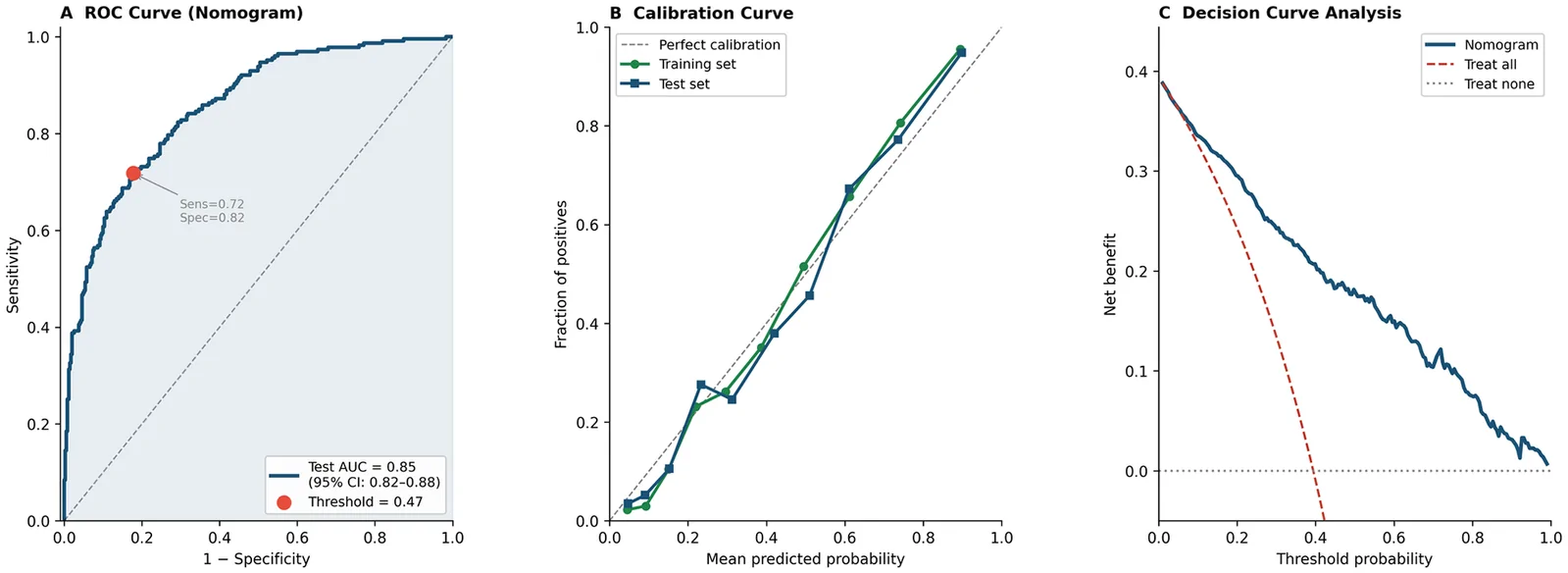
Discrimination, calibration and decision curve analysis of the nomogram on the held-out internal test set. **(A)** Receiver operating characteristic curve (AUC 0.85, 95% CI 0.82–0.88); the marked point corresponds to the Youden reference threshold of 0.47. **(B)** Calibration curves for the training and test sets against the line of perfect calibration. **(C)** Decision curve analysis showing the estimated net benefit of the nomogram relative to treat-all and treat-none strategies across threshold probabilities.

## Discussion

In this large single-centre retrospective cohort of 1,918 AC patients, we developed and internally validated a dual-framework prediction system. The ML framework reproduces three-class TG18 severity grading with macro-AUC 0.84, but balanced accuracy of only 0.65, using seven routinely available admission variables. The nomogram provides a clinician-friendly graphical tool for binary triage (Grade I vs. Grade II/III) with AUC 0.85 and potential net benefit on decision curve analysis. Together, these two frameworks address different clinical needs: the ML model supports comprehensive severity stratification, while the nomogram offers a practical bedside aid at the Grade I versus Grade II/III boundary, where TG18 recommends escalation from conservative management to early or urgent biliary drainage (1, 6). We regard the nomogram, which condenses a multi-parameter assessment into a single interpretable score at the principal decision point, as the more pragmatic contribution of this work; the multiclass ML framework primarily demonstrates the extent to which the full three-tier grading can be reproduced from seven admission variables.

An important conceptual consideration is the relationship between the predictors and the TG18-defined outcome. Several selected predictors—age, total bilirubin, albumin, platelet count, creatinine and INR—are closely related to, or are components of, the TG18 severity criteria. The models therefore partially reconstruct the reference classification from information that also informed it, rather than predicting a clinically independent outcome, and the discrimination reported here should be interpreted in that light. We do not regard this as rendering the exercise redundant, for three reasons. First, formal TG18 grading requires the integration of multiple criteria across organ systems and is inconsistently applied in routine practice (5); a deterministic score computed automatically from seven to eight routinely available admission variables can standardise that assessment, permit automated flagging within electronic health records, and serve as a second reader where hepatobiliary expertise is not immediately available. Second, the parsimonious variable set allows an estimate to be produced when some formal TG18 items have not yet been documented. Third, the performance ceiling we observe—particularly the balanced accuracy of 0.65 and the Grade II recall of 0.56—quantifies how much of the TG18 classification is recoverable from admission laboratory data alone, which is itself informative for the design of future outcome-based models. It nevertheless remains the case that this study demonstrates agreement with TG18 categories rather than improvement in patient management or prediction of clinical outcomes. Demonstrating clinical utility would require prospective evaluation, for example a pragmatic implementation study comparing triage decisions, time to drainage, and rates of misclassification at the Grade I/II boundary with and without model support.

Our findings are consistent with and extend prior work. Existing prediction models in AC have largely addressed a dichotomised severe-versus-non-severe endpoint (9, 10) or downstream outcomes, chiefly mortality (20, 21); Lee (22) reviewed the diagnosis and severity classification of acute cholangitis. The specific contributions of the present study are the prediction of the full three-tier TG18 grading rather than a dichotomised endpoint, the pairing of that framework with an interpretable bedside nomogram at the Grade I versus Grade II/III decision point, and SHAP-based interpretability. We make no claim of primacy for the application of machine learning to AC severity prediction. Thrombocytopenia is an established marker of sepsis-induced coagulopathy and organ dysfunction (23), and a low admission platelet index has been associated with bacteraemia in acute cholangitis specifically (24); hypoalbuminaemia reflects both nutritional reserve and the systemic inflammatory response, and a serum albumin at or below 2.8 g/dL has been identified as an independent predictor of mortality and of refractory disease in acute cholangitis managed under the Tokyo Guidelines (25); composite albumin-based indices have since been associated with 30-day mortality in septic cholangitis (26). SHAP makes the contribution of each variable to an individual prediction inspectable, which may help a clinician judge when the output is worth acting on (18).

A key limitation is Grade II recall of 0.56 in the ML framework. Grade II AC is clinically heterogeneous and shares overlapping features with Grade I. The two directions of misclassification carry different consequences: the 25% of Grade II patients assigned to Grade I would risk delayed drainage, whereas the 19% assigned to Grade III would risk unnecessary escalation of care. Because improving recognition of moderate disease was a principal motivation for this work, the multiclass framework in its present form does not achieve clinically acceptable Grade II performance, and its output should not be used in isolation to withhold or defer drainage. Future work should explore Grade II-specific oversampling strategies, ordinal rather than nominal modelling of severity, or ensemble methods. A second limitation is the single-centre retrospective design. All performance estimates derive from one tertiary hepatobiliary centre in China; external validation in multicentre and international cohorts is required. Third, TG18 grade served as the surrogate endpoint; future studies should validate these models against hard clinical outcomes such as 30-day mortality and emergency intervention rate. Fourth, the Youden index weights sensitivity and specificity equally, which is not appropriate when failing to recognise Grade II/III disease is more costly than unnecessary escalation; the multi-threshold analysis (Supplementary Table S9) and the decision-curve framework should therefore guide threshold selection in a given setting, and the rule-out threshold of 0.20 may be preferable where missed moderate-to-severe disease is the dominant concern. Fifth, although overall missingness was low, total cholesterol and low-density lipoprotein cholesterol were missing in 14.6% of patients; neither entered the final models, and procalcitonin was excluded *a priori* for the same reason. Sixth, the exclusion of patients meeting only the TG18 criteria for a suspected diagnosis means that the models have not been evaluated in the diagnostically ambiguous group in which decision support might be of greatest value.

Prospective multicentre validation, preferably incorporating at least one non-Chinese cohort, is required before clinical deployment of either framework.

## Conclusion

We present a dual-framework system combining ML-based three-class TG18 severity grading and a logistic regression nomogram for binary triage of acute cholangitis, developed and internally validated on 1,918 patients from a single centre. Discrimination was good for both frameworks and the nomogram showed potential net benefit on decision curve analysis, but recognition of Grade II disease by the multiclass model remained limited (recall 0.56, balanced accuracy 0.65). Because both models predict the TG18 grade itself, they are best understood as tools for standardising severity assessment rather than as independent predictors of clinical outcome. Prospective external validation against clinical outcomes is required before clinical application.

## Data Availability

The raw data supporting the conclusions of this article will be made available by the authors, without undue reservation.
