# Supplementary material for: Machine learning and nomogram for predicting severity of acute cholangitis: development and internal validation of a dual-framework model in a retrospective cohort study
Source: Front Med (Lausanne). 2026 Oct 6;13:1936260. doi: 10.3389/fmed.2026.1936260 (PMC13693306; doi:10.3389/fmed.2026.1936260)
Supplement: Supplementary file 1 [file Datasheet1.docx]

**Supplementary Materials**

Machine Learning and Nomogram for Predicting Severity of Acute Cholangitis: Development and Internal Validation of a Dual-Framework Model in a Retrospective Cohort Study

*Manuscript ID 1936260 — revised version (R1). This document supersedes and replaces the Supplementary Materials submitted with the original manuscript.*

**Contents**

Supplementary Methods — Extended statistical and modelling details

Table S1 — Univariate Kruskal–Wallis screening of all 18 prespecified candidate predictors

Table S2 — Collinearity assessment and variable-removal decisions

Table S3 — Hyperparameter search space, optimisation procedure and final values (revised)

Table S4 — Per-class one-vs-rest AUC (held-out internal test set)

Table S5 — Complete multiclass performance metrics (revised: per-class precision, recall, F1, support, balanced accuracy, confusion-matrix counts)

Table S6 — LASSO nomogram coefficients, odds ratios and 95% CIs

Table S7 — Sensitivity analysis: LASSO applied directly to all 18 candidates without pre-screening (new)

Table S8 — Leakage-controlled re-analysis with preprocessing, resampling and tuning nested within cross-validation folds (new)

Table S9 — Nomogram operating characteristics across candidate thresholds (new)

Table S10 — Per-variable missingness among candidate predictors (new)

Table S11 — Variables retained by LASSO itself and the performance of models built on them (new)

Figure S1 — Univariate Kruskal–Wallis screening

Figure S2 — Spearman collinearity heatmap

Figure S3 — LASSO regularisation paths

Checklists — TRIPOD+AI checklist (primary); STROBE checklist (observational cohort component)

Note: the SHAP interpretability analysis previously presented as Supplementary Figure S4 has been promoted to the main text as Figure 4, and is no longer duplicated here.

**Supplementary Methods**

**S1. Candidate predictors and data preprocessing**

The source dataset comprised 1,918 patients and 27 continuous laboratory or demographic variables plus sex. Eighteen continuous variables were prespecified as candidate predictors across six clinical domains reflecting the physiology captured by the TG18 severity criteria. Sex was not a candidate predictor and was tabulated for baseline description only:

• Demographic: Age

• Hepatobiliary / cholestasis: TB, DB, ALB, ALT, AST, ALP, GGT

• Systemic inflammation: WBC, NEU, CRP

• Coagulation: PT, INR, PLT

• Renal function: SCr, BUN

• Metabolic / nutritional: TC, LDL

Procalcitonin was excluded a priori because 36.6% of values were missing, exceeding the prespecified 20% threshold for reliable imputation. Variables not prespecified as candidates (lymphocyte and monocyte counts, haemoglobin, haematocrit, calcium, fasting blood glucose, triglycerides, high-density lipoprotein cholesterol) were not entered into any model. Sex, comorbidities, aetiology and hospital length of stay were tabulated for baseline description only (Table 1); none entered any model.

All predictor values were the first recorded after presentation, obtained within 24 h of admission and before antibiotic administration. These are the same admission values that were available to the attending team when the TG18 reference grade was assigned; the resulting shared information between predictors and outcome is discussed explicitly in the main text.

Missing values were imputed by median imputation. Imputation parameters were estimated on the training set only and applied unchanged to the test set. Feature distributions were examined graphically. Predictors were standardised (z-scored) before penalised regression so that the LASSO penalty applied comparably across variables on different scales; no further transformation was applied. Tree-based models are unaffected by monotone transformation of a predictor, but penalised logistic regression is not, and leaving skewed predictors untransformed constrains the nomogram to a linear relationship on the log-odds scale. This is a modelling assumption rather than a neutral choice, and we note it as such.

**S2. Three-step variable selection**

Step 1 — Univariate screening. Kruskal–Wallis tests were applied to all 18 candidates across the three TG18 grades. The tests were computed on the training set alone (n = 1,342), so that no information from the held-out test set could influence which variables were carried forward; a deliberately liberal threshold of P < 0.10 was used to avoid premature exclusion. Two candidates failed screening — ALT (H = 2.7, P = 0.256) and AST (H = 4.0, P = 0.138) — leaving 16 variables (Table S1).

Step 2 — Collinearity. Spearman correlations were computed among retained variables; where |r| ≥ 0.80, the member of the pair with the weaker univariate association was removed to avoid redundant assays of the same biological construct (Table S2).

Step 3 — LASSO. LASSO regularisation with tenfold cross-validated penalty selection was applied on the training set, separately for the multiclass and binary frameworks (Figure S3), yielding seven and eight variables respectively.

Because univariate pre-screening before penalised multivariable modelling can in principle discard predictors that are informative only in a multivariable context, a sensitivity analysis applying LASSO directly to all 18 prespecified candidates, without screening or collinearity filtering, is reported in Table S7.

**S3. Model development, resampling and leakage control**

The cohort was split 70/30 (training n = 1,342; held-out internal test n = 576), stratified by TG18 grade, with random seed 42. The split preceded all preprocessing, and the test set was used only for final evaluation.

In the primary analysis, median imputation, standardisation, variable selection and SMOTE were each fitted on the whole training set, and five-fold stratified cross-validation of the training set was used to select hyperparameters. SMOTE was applied to training data only; the held-out test set was never resampled, so the reported test-set estimates cannot be inflated by synthetic observations. Fitting preprocessing and resampling on the full training set before cross-validation can, however, render hyperparameter selection mildly optimistic. Two confirmatory analyses therefore encapsulated median imputation, standardisation, SMOTE and the classifier in a single pipeline that was refitted independently within every cross-validation training fold: the first held the seven selected variables fixed, and the second additionally refitted the LASSO selection step within each fold, applied to the 16 variables retained after screening. The univariate Kruskal–Wallis screening and the collinearity filtering that preceded LASSO were carried out once on the whole training set and were not repeated within folds. These analyses therefore bound the optimism attributable to preprocessing, resampling, hyperparameter tuning and the LASSO step, but do not eliminate the possibility of a small residual contribution from the earlier screening steps; we do not claim a fully nested variable-selection procedure (Table S8).

Analyses were performed in Python 3.10 (scikit-learn, imbalanced-learn, xgboost, shap) and R 4.3.2 (glmnet, rms). Random seed 42 was used throughout. Bootstrap confidence intervals were derived from 1,000 iterations.

**Supplementary Table S1. Univariate Kruskal–Wallis screening of all 18 prespecified candidate predictors (training set, n = 1,342)**

| **Variable** | **Kruskal–Wallis H** | **P value** | **Missing (%)** | **Decision** |
| --- | --- | --- | --- | --- |
| ALB | 276.4 | <0.001 | 0.00 | Retained |
| INR | 234.2 | <0.001 | 2.45 | Retained |
| Age | 210.4 | <0.001 | 0.00 | Retained |
| PLT | 201.8 | <0.001 | 0.83 | Retained |
| CRP | 170.2 | <0.001 | 4.64 | Retained |
| PT | 166.6 | <0.001 | 2.45 | Retained |
| TC | 157.9 | <0.001 | 14.60 | Retained |
| LDL | 152.6 | <0.001 | 14.60 | Retained |
| NEU | 124.4 | <0.001 | 0.83 | Retained |
| BUN | 113.3 | <0.001 | 1.98 | Retained |
| DB | 111.4 | <0.001 | 0.00 | Retained |
| WBC | 86.1 | <0.001 | 0.83 | Retained |
| TB | 82.6 | <0.001 | 0.00 | Retained |
| SCr | 71.2 | <0.001 | 1.09 | Retained |
| ALP | 33.5 | <0.001 | 0.00 | Retained |
| GGT | 9.1 | 0.0105 | 0.00 | Retained |
| AST | 4.0 | 0.138 | 0.00 | Excluded (P ≥ 0.10) |
| ALT | 2.7 | 0.256 | 0.00 | Excluded (P ≥ 0.10) |

Tests were computed on the training set only (n = 1,342), before any model fitting, so that variable selection was not informed by the held-out test set; the missingness column refers to the full cohort. The prespecified retention threshold was P < 0.10. Procalcitonin was excluded a priori (36.6% missing) and is not shown.

**Supplementary Table S2. Collinearity assessment on the training set and variable-removal decisions**

| **Variable pair** | **Spearman r (training set)** | **Retained** | **Basis for the choice** |
| --- | --- | --- | --- |
| Total bilirubin — direct bilirubin | +0.976 | Total bilirubin | Reported by every routine liver panel; no missing values in this cohort |
| White cell count — neutrophil count | +0.972 | Neutrophil count | Stronger univariate association with grade (H = 124.4 vs 86.1) |
| Prothrombin time — INR | +0.847 | INR | Standardised across laboratories; stronger univariate association (H = 234.2 vs 166.6) |
| Total cholesterol — LDL cholesterol | +0.938 | Total cholesterol | Stronger univariate association (H = 157.9 vs 152.6); neither entered the final models |

Spearman correlations were computed on the training set only (n = 1,342). Where |r| ≥ 0.80 the pair was treated as measuring the same construct and only one member was carried forward, so that the resulting tools would not require duplicate assays; the choice was made on clinical availability where the two were interchangeable, and otherwise on the stronger univariate association. Twelve variables entered LASSO. The full correlation matrix is shown in Figure S2.

**Supplementary Table S3 (revised). Hyperparameter search space, optimisation procedure and final selected values**

| **Model** | **Search space** | **Final values (primary analysis)** | **Final values (pipeline refitted within folds)** |
| --- | --- | --- | --- |
| Random forest | n_estimators {200, 500}; max_depth {None, 6, 10}; min_samples_leaf {1, 2, 5} (18 combinations) | n_estimators = 500; max_depth = None; min_samples_leaf = 2 | n_estimators = 500; max_depth = 10; min_samples_leaf = 2 |
| XGBoost | n_estimators {200, 500}; max_depth {3, 6}; learning_rate {0.05, 0.1} (8 combinations) | n_estimators = 200; max_depth = 6; learning_rate = 0.1 | n_estimators = 200; max_depth = 3; learning_rate = 0.05 |
| SVM (RBF kernel) | C {0.1, 1, 10}; gamma {scale, 0.1} (6 combinations) | C = 1.0; gamma = scale | C = 0.1; gamma = 0.1 |

Optimisation metric: macro one-vs-rest AUC under five-fold stratified cross-validation of the training set (n = 1,342). Grid search was exhaustive over the stated space. The final column reports the values selected when median imputation, standardisation, SMOTE and the classifier were refitted within every cross-validation fold (Table S8). The univariate screening and collinearity filtering that precede these steps were carried out once on the training set and were not repeated within folds, so this is not a fully nested procedure; performance under the two sets of hyperparameters was nevertheless equivalent.

**Supplementary Table S4. Per-class one-vs-rest AUC on the held-out internal test set (n = 576)**

| **Model** | **Grade I** | **Grade II** | **Grade III** | **Macro AUC** | **95% CI** |
| --- | --- | --- | --- | --- | --- |
| Random forest | 0.891 | 0.797 | 0.839 | 0.843 | 0.812–0.870 |
| XGBoost | 0.892 | 0.804 | 0.817 | 0.838 | 0.807–0.866 |
| SVM (RBF) | 0.866 | 0.747 | 0.801 | 0.804 | 0.772–0.836 |

**Supplementary Table S5 (revised). Complete multiclass performance metrics on the held-out internal test set (n = 576)**

| **Model** | **Grade** | **AUC (OvR)** | **Precision** | **Recall (sensitivity)** | **F1-score** | **Support (n)** |
| --- | --- | --- | --- | --- | --- | --- |
| Random forest | Grade I | 0.891 | 0.863 | 0.828 | 0.845 | 349 |
|  | Grade II | 0.797 | 0.468 | 0.559 | 0.510 | 118 |
|  | Grade III | 0.839 | 0.610 | 0.560 | 0.584 | 109 |
| Random forest — overall | — | Macro AUC 0.843 | Accuracy 72.2% | Balanced acc. 0.649 | Macro F1 0.646 | 576 |
| XGBoost | Grade I | 0.892 | 0.845 | 0.842 | 0.844 | 349 |
|  | Grade II | 0.804 | 0.473 | 0.517 | 0.494 | 118 |
|  | Grade III | 0.817 | 0.596 | 0.541 | 0.567 | 109 |
| XGBoost — overall | — | Macro AUC 0.838 | Accuracy 71.9% | Balanced acc. 0.634 | Macro F1 0.635 | 576 |
| SVM (RBF) | Grade I | 0.866 | 0.873 | 0.731 | 0.796 | 349 |
|  | Grade II | 0.747 | 0.384 | 0.517 | 0.440 | 118 |
|  | Grade III | 0.801 | 0.488 | 0.560 | 0.521 | 109 |
| SVM (RBF) — overall | — | Macro AUC 0.804 | Accuracy 65.5% | Balanced acc. 0.602 | Macro F1 0.586 | 576 |

Balanced accuracy is the unweighted mean of per-class recall, reported because overall accuracy is dominated by the majority Grade I class.

**Confusion matrices (absolute counts; rows = true grade, columns = predicted grade)**

**Random forest**

| **True \ Predicted** | **Grade I** | **Grade II** | **Grade III** | **Total** |
| --- | --- | --- | --- | --- |
| Grade I | 289 | 44 | 16 | 349 |
| Grade II | 29 | 66 | 23 | 118 |
| Grade III | 17 | 31 | 61 | 109 |

**XGBoost**

| **True \ Predicted** | **Grade I** | **Grade II** | **Grade III** | **Total** |
| --- | --- | --- | --- | --- |
| Grade I | 294 | 39 | 16 | 349 |
| Grade II | 33 | 61 | 24 | 118 |
| Grade III | 21 | 29 | 59 | 109 |

**SVM (RBF)**

| **True \ Predicted** | **Grade I** | **Grade II** | **Grade III** | **Total** |
| --- | --- | --- | --- | --- |
| Grade I | 255 | 61 | 33 | 349 |
| Grade II | 26 | 61 | 31 | 118 |
| Grade III | 11 | 37 | 61 | 109 |

For the random forest, 29 of 118 Grade II patients (25%) were classified as Grade I and 23 (19%) as Grade III. The former direction risks delayed drainage and the latter unnecessary escalation of care; both are discussed in the main text.

**Supplementary Table S6. LASSO nomogram coefficients, odds ratios and 95% CIs (Grade I versus Grade II/III)**

| **Rank** | **Variable** | **LASSO coefficient** | **Odds ratio (per 1 SD)** | **95% CI** | **Association with Grade II/III risk** |
| --- | --- | --- | --- | --- | --- |
| 1 | Age (years) | +0.557 | 1.75 | 1.50–2.02 | Increases risk |
| 2 | INR | +0.483 | 1.62 | 1.20–2.13 | Increases risk |
| 3 | Albumin (g/L) | −0.452 | 0.64 | 0.55–0.74 | Decreases risk (protective) |
| 4 | Platelet count (×10⁹/L) | −0.427 | 0.65 | 0.56–0.76 | Decreases risk (protective) |
| 5 | Total bilirubin (µmol/L) | +0.414 | 1.51 | 1.30–1.76 | Increases risk |
| 6 | Neutrophil count (×10⁹/L) | +0.365 | 1.44 | 1.24–1.68 | Increases risk |
| 7 | C-reactive protein (mg/L) | +0.223 | 1.25 | 1.07–1.46 | Increases risk |
| 8 | Serum creatinine (µmol/L) | +0.213 | 1.24 | 1.05–1.46 | Increases risk |

LASSO penalised logistic regression with tenfold cross-validated penalty applied to standardised (z-scored) predictors; coefficients are log-odds per one standard deviation increase. Odds ratios and 95% CIs were estimated by 1,000 bootstrap iterations on the training set (n = 1,342). Selected penalty C = 0.042.

**Supplementary Table S7 (new). Sensitivity analysis — LASSO applied directly to all 18 prespecified candidates, without univariate screening or collinearity filtering**

| **Framework** | **Variables selected** | **Test-set performance** | **Primary (parsimonious) model** |
| --- | --- | --- | --- |
| Binary — Grade I vs Grade II/III | INR (+0.77), Age (+0.66), PLT (-0.50), NEU (+0.43), ALB (-0.40), TB (+0.34), CRP (+0.25), DB (+0.25), SCr (+0.24), BUN (+0.16), TC (-0.14), PT (+0.09), GGT (+0.04), ALT (-0.04), ALP (+0.04) | AUC 0.858 | 8-variable nomogram: AUC 0.855 |
| Multiclass — Grade I/II/III | Age, TB, DB, ALB, ALP, NEU, PLT, PT, INR, SCr, BUN, CRP, TC (13 variables) | Macro AUC 0.846 | 7-variable random forest: macro AUC 0.843 |

Binary framework: LASSO logistic regression with tenfold cross-validated penalty on all 18 candidates; coefficients shown in parentheses as log-odds per standard deviation, ranked by absolute magnitude. Multiclass framework: multinomial LASSO selection followed by a random forest refitted on the selected variables. Discrimination differed from the primary models by ≤0.01 AUC in both frameworks, and the additional variables carried only marginal coefficients, indicating that the sequential selection strategy did not discard materially informative predictors while yielding a substantially more parsimonious bedside tool.

**Supplementary Table S8 (new). Leakage-controlled re-analysis with preprocessing, resampling and tuning nested within cross-validation folds**

| **Model** | **Primary analysis — test macro AUC** | **Nested pipeline (fixed variables) — CV macro AUC (mean ± SD)** | **Nested pipeline (fixed variables) — test macro AUC** | **Nested pipeline + LASSO step refitted in-fold — test macro AUC** |
| --- | --- | --- | --- | --- |
| Random forest | 0.843 | 0.873 ± 0.009 | 0.846 | 0.851 |
| XGBoost | 0.838 | 0.872 ± 0.012 | 0.847 | 0.858 |
| SVM (RBF) | 0.804 | 0.841 ± 0.011 | 0.821 | 0.822 |

In both analyses, median imputation, standardisation, SMOTE and the classifier were encapsulated in a single imbalanced-learn pipeline refitted independently within each cross-validation training fold, with hyperparameters tuned inside the same folds. In the final column the LASSO selection step was additionally refitted within each fold, applied to the 16 variables retained after screening; the univariate screening and collinearity filtering that preceded it were performed once on the whole training set and were not repeated within folds. These analyses therefore quantify the optimism attributable to preprocessing, resampling, hyperparameter tuning and LASSO selection, and do not claim a fully nested variable-selection procedure. Test-set macro AUCs match or marginally exceed the primary analysis, indicating that these steps contributed no material optimism. Because the univariate screening and collinearity filtering were not repeated within folds, the analyses bound rather than exclude optimism arising from those earliest steps. The held-out test set was never resampled in any analysis.

**Supplementary Table S9 (new). Nomogram operating characteristics across candidate probability thresholds (held-out internal test set)**

| **Threshold** | **Sensitivity** | **Specificity** | **PPV** | **NPV** | **False negatives (n)** | **Missed cases per 100 patients with Grade II/III** | **Missed cases per 100 patients tested** | **Net benefit** |
| --- | --- | --- | --- | --- | --- | --- | --- | --- |
| 0.20 (rule-out) | 0.952 | 0.476 | 0.541 | 0.938 | 11 | 4.8 | 1.9 | 0.296 |
| 0.30 | 0.859 | 0.628 | 0.600 | 0.873 | 32 | 14.1 | 5.6 | 0.242 |
| 0.40 | 0.793 | 0.734 | 0.659 | 0.845 | 47 | 20.7 | 8.2 | 0.205 |
| 0.47 (Youden) | 0.714 | 0.822 | 0.723 | 0.815 | 65 | 28.6 | 11.3 | 0.186 |
| 0.50 | 0.683 | 0.851 | 0.749 | 0.805 | 72 | 31.7 | 12.5 | 0.179 |
| 0.60 | 0.546 | 0.928 | 0.832 | 0.759 | 103 | 45.4 | 17.9 | 0.150 |
| 0.70 | 0.396 | 0.963 | 0.874 | 0.710 | 137 | 60.4 | 23.8 | 0.104 |

Test set n = 576, of whom 227 had Grade II/III disease. The two 'missed cases' columns use different denominators and are reported separately to avoid ambiguity: the first is 100 × (1 − sensitivity), i.e. the number missed among 100 patients who actually have Grade II/III disease; the second is 100 × false negatives / total tested, i.e. the number missed among 100 consecutive patients assessed.

Net benefit is calculated as (true positives − false positives × [p/(1 − p)]) / n at each threshold probability p, as in Figure 6C. The Youden threshold weights sensitivity and specificity equally, which is not appropriate when failure to recognise moderate-to-severe disease is the dominant clinical concern; the rule-out threshold of 0.20 is offered for that setting. Threshold choice should be tailored to the local consequence ratio.

**Supplementary Table S10 (new). Per-variable missingness among candidate predictors before imputation (n = 1,918)**

| **Variable** | **Missing (%)** | **Handling** |
| --- | --- | --- |
| PCT (excluded a priori) | 36.55 | Excluded a priori (>20% threshold); never entered any model |
| TC | 14.60 | Median imputation; not retained by LASSO in any final model |
| LDL | 14.60 | Median imputation; not retained by LASSO in any final model |
| CRP | 4.64 | Median imputation |
| PT | 2.45 | Median imputation |
| INR | 2.45 | Median imputation |
| BUN | 1.98 | Median imputation |
| SCr | 1.09 | Median imputation |
| WBC | 0.83 | Median imputation |
| NEU | 0.83 | Median imputation |
| PLT | 0.83 | Median imputation |

Overall, 2.5% of all candidate feature values were missing. Sex was missing in 0.99% of patients. Variables not listed had no missing values (age, total bilirubin, direct bilirubin, albumin, ALT, AST, ALP, GGT). Imputation medians were estimated on the training set and applied unchanged to the test set. No patient was excluded on the basis of missingness in these variables.

**Supplementary Table S11 (new). Variables retained by LASSO itself, and the performance of models built on them**

The final models were restricted to the variables with the largest LASSO coefficients that are also routinely available on admission, so that the bedside tool would remain short. Because that last step was taken for parsimony rather than by a statistical rule, the full LASSO solutions and their performance are reported here. The seven and eight variables used in the paper are a subset of every solution below; no variable used in the final models was rejected by LASSO.

| **Selection rule** | **Variables retained** | **n** |
| --- | --- | --- |
| LASSO, one-standard-error rule | Age, TB, ALB, NEU, PLT, INR, SCr, BUN, CRP, TC | 10 |
| LASSO, cross-validated optimum | Age, TB, ALB, ALP, GGT, NEU, PLT, INR, SCr, BUN, CRP, TC | 12 |
| Used in the three-class ML framework | ALB, INR, Age, PLT, NEU, TB, SCr | 7 |
| Used in the binary nomogram | ALB, INR, Age, PLT, NEU, TB, SCr, CRP | 8 |

**Performance on the held-out internal test set (n = 576)**

| **Variable set** | **Three-class macro AUC** | **Accuracy** | **Balanced accuracy** | **Grade II recall** | **Nomogram AUC** |
| --- | --- | --- | --- | --- | --- |
| 7 / 8 variables (used in the paper) | 0.843 | 0.722 | 0.649 | 0.559 | 0.855 |
| 10 variables (one-standard-error rule) | 0.850 | 0.720 | 0.652 | 0.568 | 0.857 |
| 12 variables (cross-validated optimum) | 0.846 | 0.724 | 0.656 | 0.568 | — |

Three-class models are random forests trained on the SMOTE-balanced training set; the nomogram is LASSO penalised logistic regression. All differences are 0.007 or less in discrimination and fall well within the bootstrap confidence interval of the primary estimate (0.812–0.870), so the parsimonious models perform neither better nor worse than the fuller ones while requiring fewer tests.
