# Supplementary material for: Machine learning and nomogram for predicting severity of acute cholangitis: development and internal validation of a dual-framework model in a retrospective cohort study
Source: Front Med (Lausanne). 2026 Oct 6;13:1936260. doi: 10.3389/fmed.2026.1936260 (PMC13693306; doi:10.3389/fmed.2026.1936260)
Supplement: Supplementary file 2 [file Datasheet2.docx]

**TRIPOD+AI Checklist**

Machine Learning and Nomogram for Predicting Severity of Acute Cholangitis: Development and Internal Validation of a Dual-Framework Model in a Retrospective Cohort Study (Manuscript ID 1936260, revised version R1)

*Items follow the TRIPOD+AI statement (Collins GS, Moons KGM, Dhiman P, Riley RD, Beam AL, Van Calster B, et al. BMJ. 2024;385:e078378; doi 10.1136/bmj-2023-078378), which supersedes TRIPOD 2015 for prediction models developed using regression or machine learning methods. Page and line numbers refer to the clean (changes-accepted) revised manuscript.*

| **Section** | **Item** | **Checklist item** | **Where addressed in the revised manuscript** |
| --- | --- | --- | --- |
| Title | 1 | Identify the study as developing or evaluating the performance of a multivariable prediction model, the target population, and the outcome to be predicted. | Title page, p.1 lines 1–3. Title revised to state 'Development and Internal Validation of a Dual-Framework Model'; target population (acute cholangitis) and outcome (TG18 severity grade) named. |
| Abstract | 2 | See TRIPOD+AI for Abstracts checklist. | Abstract, pp.1–2 lines 18–50. Reports design, setting, participants, sample size, predictors, outcome, analysis, performance including per-class recall, and limitations. |
| Introduction | 3a | Explain the healthcare context and rationale for developing or evaluating the model, including references to existing models. | Introduction, p.2 lines 45–63; existing binary models cited (refs 20, 22). |
|  | 3b | Describe the objectives, including whether the study describes development, evaluation, or both. | Introduction, p.2 lines 64–77. Development with internal validation; both frameworks specified. |
| Methods | 4a | Describe the sources of data, separately for development and evaluation datasets. | Methods — Study Design and Patients, p.2 lines 72–77. Single-centre electronic medical records, 2021–2025. |
|  | 4b | Specify the dates of the collected participant data. | Methods, p.2 lines 75–76 (January 2021 – December 2025). |
|  | 5a | Specify key elements of the study setting. | Methods, p.2 lines 73–74 (tertiary hepatobiliary referral centre, Zhejiang, China). |
|  | 5b | Describe eligibility criteria for participants. | Methods, p.2 lines 78–86. Revised to reproduce the TG18 definite diagnostic criteria (domains A + B + C); suspected-diagnosis patients excluded. |
|  | 5c | Give details of any treatments received. | Methods, p.3 lines 98–102. All predictors drawn before antibiotic administration; drainage decisions not part of the modelled outcome. |
|  | 6 | Clearly define the outcome that is predicted, including how and when assessed. | Methods — Variables and Outcome, p.3 lines 98–102. TG18 severity grade assigned by the attending team within 24 h of admission. |
|  | 7 | Describe predictors used, including how and when measured; state any pre-processing. | Methods, p.3 lines 103–118. Eighteen prespecified candidates across six domains; first values within 24 h of admission; median imputation and standardisation described. |
|  | 8 | Explain how missing data were handled. | Methods — Missing Data, p.4 lines 166–177; Supplementary Table S10 (per-variable missingness). |
|  | 9 | Describe how the data were used (e.g. training, tuning, evaluation) and any data partitioning. | Methods, p.2 lines 89–92; Supplementary Methods S3. Stratified 70/30 split before preprocessing, seed 42; test set used only for final evaluation. |
|  | 10 | Specify the model type, rationale, all model-building steps including any predictor selection, and method of internal validation. | Methods — Variable Selection and Machine Learning Models, p.3 lines 115–144; Supplementary Tables S3, S7, S8. |
|  | 11 | Describe how any heterogeneity in predictor effects or baseline risk was handled. | Not applicable — single-centre dataset; no clustering by site. Stated as a limitation, p.6 lines 272–275. |
|  | 12 | Specify all measures used to assess model performance and, if relevant, to compare multiple models. | Methods, p.3 lines 131–144 and p.4 lines 154–165. Macro and per-class AUC, accuracy, balanced accuracy, precision, recall, F1, calibration, decision curve analysis. |
|  | 13 | Describe any model updating arising from the evaluation. | Not applicable — no model updating was performed. |
|  | 14 | For risk groups, specify how and why they were created. | Methods, p.4 lines 158–162. Binary risk grouping (Grade I vs Grade II/III) prespecified at the TG18 drainage decision point; thresholds reported in Supplementary Table S9. |
|  | 15 | Provide details on approaches used to assess fairness, and justify choices. | Not formally assessed. Performance by sex and age stratum was not evaluated; acknowledged as a limitation. |
| Open science | 16a–16e | Funding; conflicts of interest; protocol; registration; data and code availability. | Funding, Conflict of Interest and Data Availability Statements. No prospective protocol or registration (retrospective study); analysis code available from the corresponding author. |
| Patient & public involvement | 17 | Report any patient or public involvement during the study. | None — retrospective analysis of routinely collected data with waiver of consent. |
| Results | 18 | Describe the flow of participants, including the number with and without the outcome and any exclusions. | Results, p.4 lines 175–178. A total of 1,918 patients were analysed (1,163 / 391 / 364 by grade); eligibility and exclusion criteria are stated in the Methods (p.2, lines 78–88). No participant-flow diagram is provided; Figure 1 presents the analysis workflow. |
|  | 19 | Report the characteristics of the participants, including the number with missing data. | Table 1; Figure 2; Supplementary Table S10. |
|  | 20 | For each analysis, report a description of the final model and how it can be used. | Figure 5 (nomogram); Supplementary Table S6 (coefficients, odds ratios); Supplementary Table S3 (final hyperparameters). |
|  | 21 | Report performance measures with confidence intervals for the model, and any comparisons. | Table 2 (per-class and overall, with bootstrap CIs), Table 3 (nomogram), Figures 3 and 6, Supplementary Tables S4, S5, S9. |
| Discussion | 22 | Give an overall interpretation of the main results. | Discussion, pp.5–6 lines 234–252. |
|  | 23 | Discuss any limitations, including non-representative samples, overfitting, and missing data. | Discussion, p.6 lines 276–293. Incorporation of predictors into the reference standard, Grade II recall, single-centre design, surrogate endpoint, threshold weighting, missingness, exclusion of suspected diagnoses. |
|  | 24 | Discuss usability of the model in the context of current practice and any implications for practice or policy. | Discussion, pp.5–6 lines 234–252 and Conclusion, pp.6–7 lines 294–300. Framed as standardisation of TG18 assessment; prospective implementation study proposed. |
|  | 25 | Give overall interpretation of results and future directions. | Conclusion, pp.6–7 lines 294–300. |

Items reported as 'not applicable' are those relating to external validation, model updating and multi-site heterogeneity, none of which was undertaken in this development and internal-validation study. Item 15 (fairness) was not formally assessed and is acknowledged as a limitation.
